# Comparative sensitivity of social media data and their acceptable use in research

**DOI:** 10.1038/s41597-022-01773-w

**Published:** 2022-10-22

**Authors:** Libby Hemphill, Angela Schöpke-Gonzalez, Anmol Panda

**Affiliations:** 1 University of Michigan, School of Information, Ann Arbor, MI 48109 USA; 2 University of Michigan, ICPSR, Ann Arbor, MI 48104 USA

**Keywords:** Ethics, Research data

## Abstract

Social media data offer a rich resource for researchers interested in public health, labor economics, politics, social behaviors, and other topics. However, scale and anonymity mean that researchers often cannot directly get permission from users to collect and analyze their social media data. This article applies the basic ethical principle of respect for persons to consider individuals’ perceptions of acceptable uses of data. We compare individuals’ perceptions of acceptable uses of other types of sensitive data, such as health records and individual identifiers, with their perceptions of acceptable uses of social media data. Our survey of 1018 people shows that individuals think of their social media data as moderately sensitive and agree that it should be protected. Respondents are generally okay with researchers using their data in social research but prefer that researchers clearly articulate benefits and seek explicit consent before conducting research. We argue that researchers must ensure that their research provides social benefits worthy of individual risks and that they must address those risks throughout the research process.

## Introduction

Researchers have used social media data in myriad ways and through different means. For instance, Twitter, Facebook, Instagram, Reddit, and Wikipedia are the top five platforms used by social media researchers for gathering data^1^. Prior work also enumerates different ways researchers procure social media data, including web scraping using Python or R, services such as NVivo, Discovertext, NodeXL, TAGS, IFTTT, Social Feed Manager, Zapier, Hydrator, WebRecorder.io, platform APIs, and even screenshots^1,2^.

Tools for providing access to social media data are evolving. For instance, in early 2022, Twitter released its no-code API, making it possible for researchers to access Twitter data without needing to have programming expertise. Twitter data is now more accessible for a wider range of researchers to study topics like social capital^3^, political agendas^4^, labor economics^5^, and public health^6^. Likewise, the launch of the Twitter Academic API has significantly expanded the scope of Twitter data that researchers can utilize, while also affording access to the full archive of tweets previously available only through the Enterprise version.

Increased data availability makes ethical questions about social media data use for research all the more pressing. For example, *should* researchers be collecting social media data? If so, when and how ought researchers use it? Legally, social media users grant broad permissions for their data to be used when they agree to platforms’ terms of service (TOS). However, users often do not actually read or understand TOS^7^, may not think of their data as public^8^, and may not realize that researchers are among that public^9,10^. Prior work finds that users actually prefer to grant explicit consent to use their data in research despite agreeing to TOS^8^, and their attitudes toward acceptable data uses depend heavily on the research context and goals^11^. How can social media researchers reconcile legal ability to use social media data and its ready availability with individuals’ preferences about their social media data being used for research?

A basic principle in research called *respect for persons* can bring clarity to how researchers can think about ethical social media data use practices. Respect for persons^12^ requires that researchers orient their practices around individuals’ perceptions of acceptable uses of data. Respect for persons is often addressed through informed consent processes that obtain explicit permission from individuals to use their data. Explicit consent serves to inform individuals of the opportunity to have data about them collected for research, and to express their preferences about their data being collected by accepting or declining participation.

Though researchers recognize that informed consent is a consideration, along with balancing risks and benefits and protecting individuals^13^, explicit consent processes with social media users are often infeasible. The scale and anonymity of social media mean that researchers often cannot directly elicit individuals’ perceptions about the acceptable uses of data they generate on social media. One exception is the Documenting the Now Project, which created “Social Humans” labels to attach explicit use permissions to content and analyses from social media^14^. Social Humans labels aim to bridge the gap between the legal permissions users grant when agreeing to platforms’ TOS and content creators’ wishes. However, this method has yet to be widely adopted, meaning even explicit use permissions like Social Humans labels cannot effectively guide researchers about how to ensure that they are respecting individuals’ preferences about use of their data.

To understand how we can realize respect for persons in the particular context of social media data research, we look to scholarship on users’ perceptions about acceptable uses of other types of sensitive yet widely available data. Prior work finds that social media data users think of their social media data similarly to how they think about widely-recognized sensitive data types^15,16^ like voter files^17^, cell phone records^18^, and large-scale surveys^19^. Comparing individuals’ acceptable use perceptions for their social media data with these other types of sensitive data opens opportunities for us to learn from existing respect-for-persons best practices aside from explicit consent developed to support sensitive data. Our survey study thus addresses the following research questions:

- RQ1: How do participants perceptions of acceptable social media data use *compare to other types* of sensitive data about them?
- RQ2: How do participants’ perceptions of acceptable social media data use relate to the *data analyst*, their *purpose* for using the data, and perceptions of *sensitivity*?

Answering RQ1 allows us to assess social media data’s relative sensitivity, and to understand whether other sensitive data types are an appropriate context from which to seek wisdom about how researchers can approach social media data use. Answering RQ2 allows us to clarify sensitivity and other variables’ relationships to acceptable use. Together, the answers to these questions provide insights for social media researchers about which best practices to follow when working with social media data to ensure respect-for-persons.

## Factors Affecting Perceptions of Acceptable Use

Prior work argues that individuals’ willingness to share their personal data is a function of: data sensitivity, who will use the data (data analyst), what users hope to gain and who who else will benefit from using the data (data use purpose), which data will be used (data type), and personal characteristics of the person sharing the data (data sharer characteristics). The following subsections review each of these factors.

### Data sensitivity: risk perception

Data sensitivity describes how risky a person perceives sharing their data to be. Prior work on consumer willingness to share data with marketers^15,16^ identifies four types of risk that may be particularly important for people’s characterization of data as sensitive: monetary, psychological, physical, and social risk. A person’s perceptions of how sensitive a type of data is–or how much risk they perceive they will incur by sharing that type of data–may inform how willing that person may be to share their data. However, while this correlation is implied by marketing literature, the existence and quality of this relationship have yet to be empirically evaluated. Further, whether a person characterizes data about themselves as sensitive is not a fixed characteristic, but rather can change according to the contexts in which data might be used and the data sharer’s personal characteristics^15,16^.

### Data analyst: known identities

Knowing who will use their data shapes data sharers’ perceptions of acceptable use. People tend to find use of their social media data by *known* data analysts more acceptable than *unknown* data analysts. While social media users try to limit who sees and uses their data to only intended audiences, their data are still often seen by unintended or unknown audiences^10^. When people learn that these unintended audiences - among them researchers - use their data, they tend to find this use less acceptable than if their data is used by intended audiences^20^.

Beyond known versus unknown data analysts, other data analyst identities can further affect sharers’ perceptions of acceptable use. For example, Gilbert and colleagues^11^ asked respondents to rate the appropriateness of their personal Facebook data’s use for research according to the discipline using it. They found that respondents were more concerned about studies in Computer Science, Gender Studies, and Psychology using their data than studies in Health Sciences. A related study about UK public health research shows that participants were much more willing to share their personal data for research by the UK’s National Health Service than with a commercial company^21^. Aside from explicitly intended audiences, people are most comfortable with their data being used by health researchers relative to other analysts. Overall, whether a data analyst is known or unknown and which discipline or professional domain they are affiliated with can affect individuals’ perceptions of acceptable use.

### Data use purpose: benefit and process

Literature suggests that individuals’ perceptions of acceptable use also vary across data use purposes like health research or marketing. For example, a study about UK public health research found that when participants were told that mandating consent could lead to selection bias and adversely impact public health research, participants were more willing to share their data without explicit consent^21^. For both health data^21–23^ and social media data^11,24^, how much participants believe that sharing their data will contribute to a purpose that will benefit society affects how acceptable they find the use of their data.

In addition to public benefits, people are more likely to find data use acceptable when it offers personal benefits like discounts or personalized service. Researchers studying public conversations about privacy controversies found that many discussants understand themselves and their data as a “product” that for-profit companies use for the purpose of making money, and in return they receive some digital service–a personal benefit^25^. Discussants found this type of data use purpose acceptable. In another example, a study of journalists’ use of social media data suggests that the more social media users want to feel heard, the more likely they are to find journalists’ use of their data acceptable. The personal benefits of receiving digital services and “feeling heard” mediated individuals’ perceptions of different data use purposes’ acceptability^26^.

Beyond general use purpose (e.g., for public health research, for marketing, for public awareness, etc.), people’s understanding of exactly *how* their social media data will be used also affects their perceptions of its acceptable use. When participants know which analysis methods and data security measures researchers will use, they feel better about their data being used for research^22^. Fiesler and Proferes^25^ found that people who indicated understanding how their social media data would be used, including for research, were less concerned about its use relative to those who were *unaware* of how their data was later used. In general, when individuals are asked explicitly for their permission and understand what research will be conducted (i.e., data use purpose), they usually agree that their social media data can be used in research^8,11^.

### Data type: keys to personal identity and social networks

In addition to who will use their data and for what purposes, sharers care about *which* specific data will be used. Two studies examined U.S. consumers’ willingness to share different types of information with marketers^15,16^. Using a nationally representative survey, they found that respondents were just as unwilling to share their credit card number, financial account details, and driver’s license information–unique identifiers or keys that are directly tied to a specific individual–as they were to share their social network profile, profile picture, and information about their friends or family. Respondents also considered their social media profile more sensitive–or risky to share–than basic demographics such as height, place of birth, and their occupation–data that when linked to other data can increase the risk that a specific individual can be identified. When they asked respondents to rate the appropriateness of uses of their personal data for research by type of data, researchers found that respondents were most concerned about researchers using their photos and videos, data about sexual habits, data about preferences and behaviours, and posts about their friends or family members^11^. These studies show that people weigh the riskiness and acceptability of sharing various types of social media data differently.

### Individual data sharer characteristics

Research reports that perceptions of acceptable data use also vary based on a data sharer’s personal characteristics. For example, studies find that how much people trust institutions–a type of data analyst–affects whether individuals are okay with those institutions using their social media data^8,11,27^. For both health data^21–23^ and social media data^11,24^, how much people trust researchers in general also affects how acceptable they find their data’s use. Studies find mixed results concerning the effects on demographic characteristics on perceptions of acceptable use. For example, Fiesler and Proferes^8^ found that demographic characteristics have no statistically significant effect on survey respondents’ attitudes toward their Twitter data being used in various types of research. In contrast, Gilbert, Vitak, and Shilton^11^ showed that gender, age, education level, and frequency of social media use have significant effects on individuals’ attitudes toward their Facebook data being used in various type of research. A comparative study of sensitivity perceptions in Brazil and the United States found that perceptions do vary based on an individual’s country of residence (i.e., Brazil or the US) and age affect their willingness to share personal data^16^. Others also reported significant effects for sex and education level on willingness to share data in their US-based survey^15^. Finally, literature shows that pre-existing attitudes toward privacy^8^ may affect perceptions of acceptable use. These data sharer characteristics can mediate the effects of the data analyst, data use purpose, and data type on individuals’ perceptions of acceptable use.

### Summary of factors affecting perceptions of acceptable use

Overall, social media users’ perceptions of of whether using their data is acceptable may be mediated by how sensitive they perceive their data to be, the data analyst, data use purpose, data type, and their personal characteristics. This web of factors shapes the challenges that researchers face in balancing respect for persons with research needs. Our study draws from existing scholarship’s insights on sensitive data and acceptable use to learn specifically about how people feel about their *social media data’s* use relative to other data types that people have indicated is sensitive to them like health records^21^ and location^28^.

## Results

To understand participants’ perceptions about acceptable social media data use, we surveyed 1018 people through Qualtrics panels and Mechanical Turk^29^. Table 6 offers summary descriptive statistics about our sample. We used statistical analyses to identify patterns in survey responses, specifically regarding (a) the *sensitivity* of individuals’ online identifiers relative to other personal identifiers, and (b) whether or not participants perceive a *particular data analyst* using a *specific type of data* for a *purpose* is acceptable or not. Section 5 provides additional details about our survey design, variables, and analysis techniques. We address each research question in its own section below. In each case, we have provided the regression models of best fit.

### RQ1: How do participants perceptions of acceptable social media data use compare to other types of sensitive data about them?

**Fig. 1:**
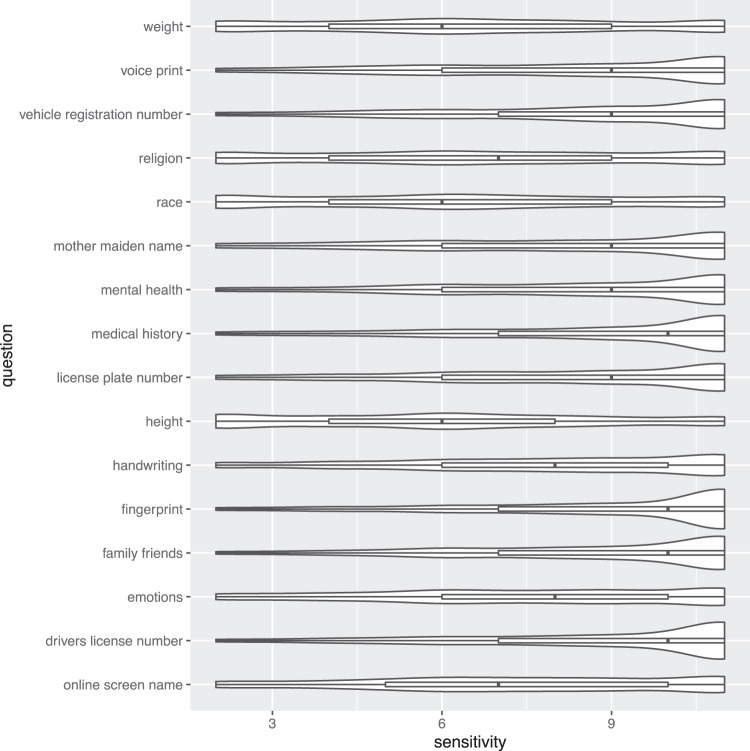
Violin plots showing the distribution, median, and quartiles for sensitivity of various types of data where 10 = ‘very sensitive’.

**Fig. 2:**
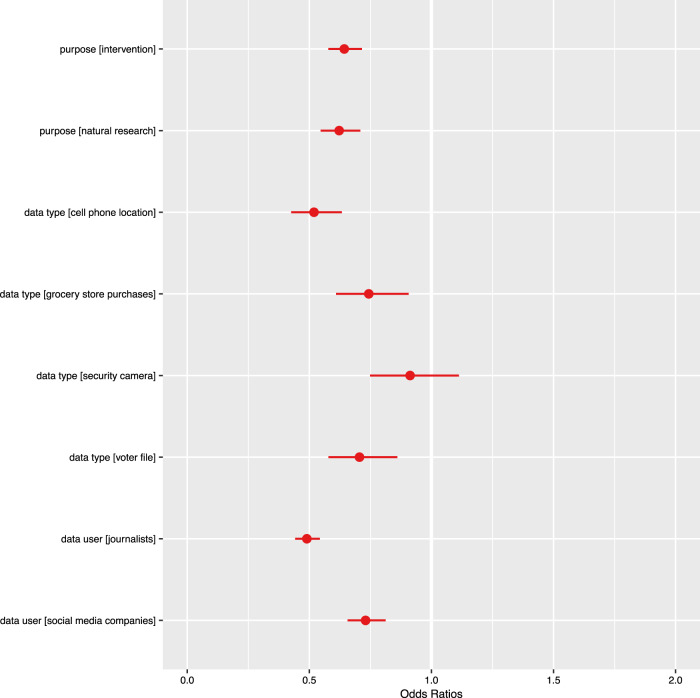
Odds ratio plot where DV = “is ok” on each data analyst, data type, purpose combination.

We calculated cumulative link mixed model (CLMM) using the ordinal package in R^30^ to compare the relative sensitivity of a key to one’s social media data - one’s screen name - with other types of similar keys to potentially sensitive data about individuals (e.g., driver’s license number or social security number). We also compare the sensitivity of one’s screen name with data that in aggregate could be linked to a specific individual’s identity (e.g., race, weight). Our results, presented in Table 1 and Fig. 1, show that a screen name is more sensitive than demographic details such as race (OR = 0.36, *p* < 0.001), religion (OR = 0.50 *p* < 0.001), and weight (OR = 0.47 *p* < 0.001), but less sensitive than identifiers such as a driver’s license number (OR = 4.67, *p* < 0.001) or data from one’s medical history (OR = 4.46, *p* < 0.001). Figure 2 also shows that individuals exhibited more variation in the sensitivity of their online screen name than other types of data such as fingerprints or medical history that may also provide access to data about their behaviors.

**Table 1:** Predicting the sensitivity of various data types; controls include demographic variables; “online screen name” is the reference category.

Variables of Interest	OR	Control Variables	OR
**Data Type**		**Recruitment Site**	
Drivers license no.	4.67***	Qualtrics	1.42***
Emotions	1.15	**Behavior Scales**	
Family friends	3.80***	Trust	0.97***
Fingerprint	5.25***	Dig. priv. concern	1.28***
Handwriting	1.71***	Priv. behav.	1.09***
Height	0.30***	**Demographics**	
License plate no.	2.55***	Man	0.85***
Medical history	4.46***	Race Other	1.38**
Mental health	3.15***	Race White	1.14
Mat. maiden name	2.35***	Straight	1.45***
Race	0.36***	Age Bracket	1.17**
Religion	0.50***	Education Level	1.27***
Vehicle registr. no.	3.10***	Income level	1.13***
Voice print	2.54***		
Weight	0.47***		

‘OR’ stands for odds ratio.

***p < 0.001; **p < 0.01; *p < 0.05.

Table 1 also indicates that respondents who were men and those with higher trust in institutions were *less* likely to find their online screen name sensitive. Respondents from Qualtrics who had higher digital privacy concerns, more privacy behaviors, were neither Black nor White, were straight, older, more educated, and/or had a higher income were *more* likely to find their online screen name sensitive.

### RQ2: How do participants’ perceptions of acceptable use relate to data type, data analyst, data use purpose, and sensitivity?

We calculated a mixed effects logistic regression (MELR) using the glmer function from lme4^31^ to understand whether respondents indicated that a particular combination of data analyst, data type, and purpose was acceptable. In this model, the dependent variable was whether respondents answered “yes” to a specific question, and the questions were the only fixed effect. We included random effects for respondent and source (Qualtrics or Mechanical Turk). The coefficient for source’s random effects was nearly zero, indicating that between-subject differences based on source could be almost entirely explained by the other variables we assessed in our model. We also included demographic controls and scales for our questions about trust, digital privacy concerns, and privacy behaviors. According to ANOVA analyses, the model of best fit did *not* include sensitivity as a predictor.

The results (see Table 2 indicate respondents found only one combination clearly unacceptable (i.e., significantly lower odds ratio):

Is it ok for journalists to use posts you’ve deleted from social media in a story about natural disasters?

**Table 2:** Is it ok for this data user to use this data type for this purpose?

Data Type + Data Use Purpose	Researchers	Journalists	SMCs
**Social media post content**			
Antiracism bots	2.506***	1.514***	2.236***
Interventions to feel better	1.763***		1.480**
Language change	3.915***	1.894***	2.803***
Misinformation	3.886***	1.358*	2.698***
Predict elections	2.205***	1.451**	1.341*
Predict risk of harming others	2.421***		1.775***
Predict risk of harm to self	1.751***		1.488***
Exhibit about protest	1.917***	1.191	1.293*
Show relevant ads	2.141***		1.825***
Recognize emotions		1.015	
**Deleted social media post content**			
Information in natural disasters	1.304*	0.738*	1.099
**Social media post timestamps**			
COVID-19 spread	3.973***	1.861***	3.145***
**Social media post location data**			
COVID-19 spread	2.784***	1.499***	2.436***
**Social media image content**			
Train facial recognition	1.198	0.885	1.038
Vegetation in national parks	3.745***	1.810***	2.251***
**Social media image metadata**			
Map of peonies	1.259	0.866	1.258
**Security camera footage**			
Train facial recognition	1.763***		1.232
**Cell phone location**			
Commuting patterns	1.449**	0.815	1.023
**Grocery store purchases**			
Send you coupons	3.386***		2.382***
Influence of other shoppers	2.316***	1.117	1.726***
**Voter file**			
Voter turnout	2.129***	1.029	1.156
**Controls**	**OR**	**Controls**	OR
**Behavior Scales**		**Demographics**	
Trust	1.041***	Straight	0.265***
Digital privacy concern	0.892**	Age brackets	0.618**
Privacy behaviors	1.112***	Man	1.980***
**Demographics**		Education level	1.356
Race other	2.116*	Income level	1.058
Num.Obs.	55049	BIC	51021.3

“No” is the reference category; ORs greater than 1 indicate respondents were more likely to say a use was “ok”. Missing cells mean that combination was not included on the survey.

***p < 0.001; **p < 0.01; *p < 0.05.

Overall, respondents said that it was *more* acceptable to use their social media data than other types of data (see Table 3).

**Table 3:** Summary of acceptable use where data type, date use purpose, and data analyst are aggregated.

Variable	Odds Ratio
**Data Type**	
Cell phone location	0.519***
Grocery store purchases	0.743**
Security camera	0.913
Voter file	0.705***
**Data Use Purpose**	
Intervention	0.643***
Natural research	0.622***
**Data Analyst**	
Journalists	0.490***
Social media companies (SMC)	0.730***
**Behavior Scales**	
Trust	1.044***
Digital privacy concern	0.880**
Privacy behaviors	1.130***
**Demographics**	
Man	1.945***
Race other	1.645
Race white	1.224
Straight	0.235***
Age brackets	0.646*
Education level	1.401
Income level	0.978
**Interactions**	**Odds Ratio**
**Data Type** × **Data Analyst**	
Cell phone location × journalists	1.161
Grocery store purchases × journalists	1.058
Security camera × journalists	0.924
Voter file × journalists	0.979
Cell phone location × SMC	0.917
Grocery store purchases × SMC	1.026
Security camera × SMC	0.828
Voter file × SMC	0.700*
**Data Use Purpose** × **Data Type**	
Intervention × grocery store purchases	2.270***
**Data Use Purpose** × **Data Analyst**	
Intervention × journalists	1.273**
Natural research × journalists	1.156
Intervention × SMC	1.204*
Natural research × SMC	1.052
**Data Use Purpose** × **Data Type** × **Data Analyst**	
Intervention × grocery store purchases × SMC	0.787
Num.Obs.	43784
BIC	40662.7

Academic researchers (data user), social media data (data type), and social research (purpose) are the reference categories.

***p < 0.001; **p < 0.01; *p < 0.05.

However, respondents generally found academic researchers using social media data about them acceptable except for two scenarios (no significant difference between acceptable and not acceptable):

- images you’ve uploaded to social media to train facial recognition software?
- metadata from your photos in social media to create a public map of peony gardens in your area?

Respondents with higher scores on the digital privacy concern scale were less likely to agree that it their data’s use is acceptable (OR = 0.892). Those who had higher institutional trust scores (OR = 1.041) and privacy behavior scores (OR = 1.112) were more likely to indicate that using their data was acceptable.

Among our demographic controls, only straight (OR = 0.265) and older respondents (OR = 0.618) were less likely to answer that it was acceptable for their data to be used. Men were significantly more likely than women and other gender identities (OR = 1.980) to say it was acceptable for their data to be used. Individuals of races other than White and Black (OR = 2.116) were also more likely to find their data’s use acceptable. We observed no significant effects for income level.

We also fit a model where we collapsed our data type and purpose variables into categories. According to ANOVA analyses, we found that the best model included data type, use purpose, data analyst, behavioral scales, and demographics but *not* sensitivity as predictors of respondents finding their social media data’s use by researchers acceptable for social/behavioral research. In this model, presented in Table 3 and Fig. 2, we use *academic researchers*, *social media data*, and *social research* as the reference categories for data analyst, data type, and purpose respectively. We included a random effect for respondent and another for source (Qualtrics or Mechanical Turk). The variance between sources was nearly zero, indicating that between-source differences could be almost entirely explained by the other variables we assessed in our model. Table 3 shows that respondents were more accepting of their data being used for social research than to generate interventions or research about the natural world.

We saw similar patterns among the control variables (trust, digital privacy, privacy behavior, and demographics) in both of our models. Individuals with concerns about digital privacy (e.g., they hesitate to provide information when its requested) were less likely to be accepting of their data being used. Individuals who generally trust institutions and governments accepted their data being used by these entities. Individuals who engaged in more privacy-protecting behaviors (e.g., removing cookies from their web browser, watching for ways to control what emails they receive) were more likely to agree that their data could be used. We find that information about how sensitive someone considers their online screen name does not provide statistically significant information about how acceptable they think a combination of data, user, and purpose are.

## Discussion

Our results suggest that individuals are generally accepting of academic researchers using social media data and that online screen names are less sensitive than many other types of data, including individual identifiers such as driver’s license numbers and medical histories, but *more* sensitive than height, weight, race, and religion. Individuals indicated that academic researchers using their data was acceptable in more scenarios than journalists or social media companies doing so. Prior work on internet fandom similarly indicated more trust for researchers than for private companies and journalists^32^.

One implication of our findings is that social media researchers can leverage the expertise and practices of researchers who use other types of sensitive or moderately sensitive data such as health records. However, one potential limitation of our study and Gilbert’s^11^ is that our survey instruments clearly communicated the user, data, and purpose. In doing so, the instruments may have been specific enough that users found these scenarios acceptable; had we asked more generally about social media researchers using their data, they may not have been as accepting. We address findings about the comparative sensitivity of social media data and the relationships between sensitivity and acceptable data use below.

### Sensitivity comparison

Prior work on social media data use in research^11,25^ examines social media data on its own rather than in the context of private and/or sensitive personal information. Our survey instrument allowed us to compare individuals’ reports about the sensitivity of different types of data so that we can understand how social media data is similar to (or dissimilar from) other data often used in research. Looking at a specific type of social media data - one’s online screen name or the “key” to accessing one’s social media data, similar to one’s “offline” name–respondents suggest that their online screen name is more sensitive than demographic characteristics (e.g., one’s race) and less sensitive than other personally-identifiable data that can be linked to an individual and her behavior (e.g., one’s driver’s license number). Respondents also found online identifiers less sensitive than mental health and health records. There was also more variation in respondents’ perceptions of their online screen name’s sensitivity relative to other types of data like fingerprints or medical history that may also provide access to data about their behaviors. This variability may indicate uncertainty among individuals or true variation in our sample, and future work could attempt to verify this distribution and its causes.

### Acceptable use comparison

To evaluate perceptions of social media data’s acceptable use relative to other widely used sensitive data types, we compared social media data with examples like cell phone data and voter files that carry varying re-identification risks. Compared to these other types of sensitive data, respondents were *most* comfortable with their social media data being used, and *least* comfortable with their cell phone location data being used. This finding resonates with prior work on location data arguing that individuals expect privacy even in public^28^, and this expectation extends to automatically collected data like location captured by cell phones and social media. Relative to social media data, respondents were also less likely to agree that it’s acceptable to use their voter file or security camera footage of them. While voter files are widely used in political science research^33,34^, researchers have made important efforts to protect data sharers’ privacy expectations including disclosure risk mitigation techniques like aggregation to avoid revealing personally identifiable information.

### Sensitivity and acceptable use

We found that sensitivity is not a good predictor of whether individuals thought it was acceptable for researchers to use social media data. This result is somewhat unexpected given prior literature that suggested sensitivity mediates acceptable use^16^. One possible explanation is that ‘online screen name’ is not a useful example of social media data to ask about. It is possible, for example, that respondents may not understand how much data can be accessed when one’s online screen name is known (e.g., one’s tweet history or one’s Reddit comment history, which may reveal other types of sensitive data like religion, mental health status, etc.). Another explanation is that for other types of data, sensitivity may predict acceptable use, but for social media data, acceptable use is a function of the data analyst, data type, and purpose of data use^11^.

Concerning data analyst, type, and use purpose, our findings echo earlier results indicating users accept their social media data being used in research when told about who will use it and for what purpose^8,11^. Our respondents were generally more accepting of researchers using their data than social media companies or journalists (see Table 2). We expect that this pattern holds because users better are able to imagine benefits from research. Given the increasing distrust of journalists in the United States^35,36^, it’s not surprising that our respondents did not welcome journalists using their data. In line with prior research about sensitive data use in health research^27^ and marketing^16^, our respondents were more comfortable sharing data with researchers looking to produce social benefit and understanding than with for-profit companies using their data for similar purposes.

Given these findings, rather than looking to similarly *sensitive* data for guidance on respect-for-persons practices with social media data, our research points us to data whose analysts have *clearly communicated their use purposes’ benefits* and *cultivated trust* among prospective sharers. In fact, for both our survey and Gilbert, *et al*.‘s^11^, articulating data use scenarios explicitly may drive much of the acceptance individuals expressed.

### Acceptable use and personal characteristics

In our results, men were more likely to report that researchers could use their data without explicit permission, and that most uses of their data were acceptable. Related prior research on individuals’ willingness to share data with marketers found that sensitivity was a function of perceived privacy controls and cultural context such as masculinity values and long-term orientation^16^. The importance of masculinity values (e.g., “ It is more important for men to have a professional career than for women.”) in predicting sensitivity may explain why we observed differences between men and other gender identities. Women and members of gender identity minorities face greater risks when engaging in social media;^37,38^ those risks may lead them to be more conservative in their data sharing beliefs. As Mikal and colleagues^39^ point out, we must carefully consider who may opt-out of using social media publicly whenever we think of social media data.

We also found that older adults and straight respondents oppose the use of their data without explicit permission when asked about research generally. In another study, Dym and Fiesler^32^ found in their survey about data from online fandom communities–majority LGBTQ spaces–and their use in research that less than ten percent of their respondents used their real names in online fandom. It is possible that LGBTQ users employ privacy-protecting strategies like pseudonyms to disconnect their online screen names from their offline identities, rendering their screen names less sensitive. It is also possible that members of marginalized demographic groups may be more willing to share their data because they seek inclusion or because they see efforts to avoid surveillance as futile. Benjamin^40^ provides a thorough discussion of the differential impacts of surveillance among racial groups, for instance.

### Realizing respect for persons in social media research

Our results have two implications for realizing respect for persons in social media research. First, whenever possible, researchers should elicit informed consent from social media users to use their data in research. However, if researchers use data only from those individuals who have provided explicit permission and who are generally accepting of their data being used in research, their data will likely skew male, younger, more educated, and less straight. While bias in data cannot be eliminated and is not inherently bad, demographically-biased social media data limit their utility for population-level studies. Because individuals are generally more comfortable with population-level research than with individual-level research^8,20^, this tension is especially important for researchers to consider. Given the burden of obtaining informed consent and the biases it introduces, when it is not possible to obtain, researchers should work to anonymize data as much as possible to reduce the risks of reidentification. Existing work such as Williams, Burnap and Sloan^20^, the AOIR Ethics Guidelines^41^, and the STEP framework^42^ provide useful tools for thinking through research processes, when and how to get consent, and how to mitigate risks to individuals.

Second, as Kass *et al*.^27^ suggest, educating the public about why research is important and why it requires their data is vital to ensuring individuals’ comfort with their data being used. As earlier studies^11,24^ and now our work show, how much data sharers trust researchers affects their perceptions of acceptable use. People who place more trust in institutions were more likely to accept their data being used. We can learn from health research that has been able to explain to individuals how and why their medical records are necessary for understanding diseases such as cancer. People now tend to find their data’s use for health research more acceptable than for other uses^11,21^.

Researchers who leverage social media data can engage in similar outreach and engagement efforts to understand individuals’ hesitations and preferences. We do not, however, suggest that researchers try to cajole or coerce potential participants. Instead, social media researchers must work to ensure that their research *does* actually provide social benefits worthy of individual risks, and that they are consequently able to articulate the significance of their work so that individuals can decide whether that benefit is worth their risks. As Sloan and colleagues^43^ argue, the principles outlined in the Belmont Report apply even to social media research, and researchers have a responsibility to ensure that individuals understand their own data, how it could be used, and the risks associated with use.

In one example, Xafis^44^ demonstrated how they helped individuals understand how their data could be used and potentially competing interests between researchers and individuals represented in data. Their research showed that individuals could understand data linkage processes and the potential trade-offs quickly. Because it is not feasible to educate each potential participant that might share their social media data, the burden of education lies on researchers collectively. Researchers need to articulate the social benefits of their work so that individuals understand why disclosure and reputation risks are worth taking; if the benefits do not outweigh the risks, researchers need to be willing to abandon or avoid particular projects.

### Contextual integrity in social media research

Our work shows that beyond considering individuals’ preferences for their data’s use under a respect for persons framework, social media researchers must also attend to the contexts in which user data are generated and used. Our results confirm that contexts in which user data are used impact individuals’ perceptions of acceptable use. Specifically, they indicate relatively less comfort with their data being used for natural research or to develop interventions than with their use in social research. These findings resonate with earlier work in online fan communities, where fans expressed concerns about the contexts in which their data could be used (e.g., articles about fandom) because their identities carry different risks (e.g., fandom vs physical communities for LGBTQ folks)^32^. It also echoes prior work about Facebook data, specifically, where users expressed comfort with their data being used to improve services but not to evaluate mental health^11^. To address concerns about mismatch between data generation and use contexts, Nissenbaum^45^ offers the principle of “contextual integrity” to refer to the challenges inherent in using data generated in one context (e.g., an online discussion) in another (e.g., a research study).

### Legal constructs that impact social media data use

Recent developments in regulations, such as the EU General Data Protection Regulation (GDPR)^46^, the Digital Services Act (DSA)^47^, and proposed bills in the United States encode ethical principles in governments’ policies. For instance, GDPR specifically requires that researchers provide privacy notices and consent documents. Similarly, the DSA demands that social media companies attend to the risks related to data’s collection and use. Researchers’ obligations under GDPR, DSA, and their analogues in other jurisdictions are not yet settled, but it is clear that researchers will have regulatory requirements to meet^48^. As these regulations begin to take effect, our work based on a respect-for-persons framework suggests that future policy efforts would benefit from soliciting public input on appropriate uses of data to balance users’ expectations and preferences with researchers’ interests and goals. A one-size-fits-all approach to data reuse policy could potentially stifle research that users found acceptable and from which society could benefit.

### Summary

We began this article with an example of increased accessibility of Twitter data and raised questions for social media researchers such as: *should* they use social media data in their research? If so, *when* and *how*? As with other large-scale data, it is not always possible to ask all prospective study participants to explicitly consent to use their social media data in research. However, through surveying individuals, our study offers a benchmark of individuals’ perceptions of their social media data’s sensitivity and acceptable use from which social media data researchers can cultivate general respect-for-persons practices. We show that people generally find their social media data moderately sensitive relative to other widely used data types. People generally find it acceptable for researchers to use their social media data but prefer that researchers clearly articulate the benefits of their work. Individuals are most concerned about *who, why*, and *which data* will be used. When these factors are clearly communicated, individuals are more likely to find their data’s use for research acceptable.

Given our findings that individuals find their social media data moderately sensitive, our study invites social media researchers to learn from well-established best practices for using sensitive data and increasing public awareness about the benefits of research with social media data. Researchers must be clear with themselves and with the public about why social media data is necessary for their work and what benefits that work provides for society, especially for the individuals who carry risks by being included in the data. By using contemporary strategies to balance social media data’s use risks to individuals with the benefits to society, social media researchers can more effectively realize respect for persons, ensuring greater public support for significant advances in research.

## Methods

To understand participants’ perceptions about acceptable social media data use, we surveyed 1018 people through Qualtrics panels and Mechanical Turk^29^. Qualtrics recruitment targeted U.S. adults who posted publicly to social media at least once per week. We also used quotas for racial identity to ensure our sample was at least 10% African American individuals and at maximum was 80% white-only. We did not use targeting in recruiting MTurk participants.

We used statistical analyses to identify patterns in survey responses, specifically regarding (a) the sensitivity of individuals’ online identifiers relative to other personal identifiers, and (b) whether or not participants perceive a *particular data analyst* using a *specific type of data* for a *purpose* as acceptable or not.

### Survey population and sample size

We used the easypower^49^ package to estimate the appropriate size of our survey sample using a significance criterion of *α* = 0.05 and power = 0.80 and determined we needed at least 426 respondents to detect significant differences in the effect of the interaction of analyst, data type, and purpose. We contracted with Qualtrics to solicit responses from 586 survey panelists. Using a Qualtrics panel enabled us to set minimum quotas for our independent variables (e.g., non-white respondents) to ensure variability. We then used the same instrument to survey 432 crowdworkers through Amazon’s Mechanical Turk. We recruited panels through both Qualtrics and Mechanical Turk to determine whether recruitment platform influenced findings. In our analyses, we include random effects for recruitment source to detect differences between the samples and their impacts on our observations^50^.

### Instrument design and variables

Our survey instrument measured relationships between acceptable use and relevant constructs like data sensitivity, and personal characteristics like trust in institutions. Table 4 summarizes the various measures we included in our instrument. The following subsections explain how we developed each measure.

**Table 4:** Variables included in our survey instrument and the surveys they were drawn from.

Variable	Measure	Source
**Acceptable Use**	Binary (y/n)	^8,11,28^
**Data Sensitivity**	10-pt. scale	^15^
**Data Sharer Char**.		
Trust in institutions	7-pt Scale/SA	^62^
Dig. privacy behav.	5-pt scale/SA	^63^
Dig. privacy concern	5-pt sclae/SA	^63^
Social media use	Binary (y/n)	^65^
Social media freq.	6-pt MCQ/SA	^65^
Income	8 levels/SA	^64^
Education	8 levels/SA	^64^
Gender	6-pt MCQ/MA	^62^
Race	9-pt MCQ/MA	^64^
Sexual Orientation	5-pt MCQ/MA	^64^
Age	Manual Entry	^64^
State of Residence	Manual Entry	^64^

MCQ/MA: Multiple Choice Questions/Multiple Answers

MCQ/SA: Multiple Choice Questions/Single Answer.

#### Acceptable use

We developed a measure of acceptable use perceptions motivated by existing work that uses scenarios. However, existing acceptable use scenarios did not explicitly vary three constructs that other literature proposes affect acceptable use perceptions (“data analyst”, “data type”, and “data use purpose”). Therefore, we developed our own set of scenarios to evaluate the effects of these three constructs (see Table 5 for a summary of how we operationalized these constructs). Specifically, we varied scenarios according to three *data analysts*: academic researchers, social media companies^21^, and journalists^26^. We varied *data types* to include social media content^15^, cell phone location^51,52^, voter file^53^, survey^33^, security camera^54^, and grocery store purchase data^55^ because they are used widely in contemporary research. We included three *data use purposes*: social/behavioral research, research about the natural world, and interventions designed to change individual behavior^11^. Scenarios describing these purposes include real-world research use such as vegetation phenology^56^, emotional contagion^57^, and consumer spending^58^. They also mirror vignettes used in prior work on attitudes toward Facebook and Twitter users’ data^8,11^ and individuals’ location data^28^. We requested binary responses rather than scale-based responses to our questions about whether data use was acceptable in a scenario because scales are more difficult for respondents to interpret and answer^59,60^.

**Table 5:** Dimensions we varied in constructing our acceptable use scenarios and prior work that addressed each variable.

Dimension	Source
**Data Analyst**	
Academic researchers	^8^
Journalists	^21^
Social media companies	^26^
**Data Type**	
Social media content	^15^
Cell phone location	^51,52^
Grocery store purchases	^55^
Security camera	^54^
Voter file	^53^
Survey data	^33^
**Data Use Purposes**	^11^
Social/behavioral research	^58^
Research abt. natural world	^56^
Interven. to change behav	^57^

**Table 6:** Overview of our survey sample’s personal characteristics.

Variables	Levels	N	%
Man	Yes	551	54%
	No	456	45%
Straight	Yes	844	83%
	No	161	16%
Race	Black	90	9%
	White	765	75%
	Other	140	14%
Age	18–24	345	34%
	35–64	398	39%
	65 and over	275	27%
Education	Less than college degree	342	34%
	College degree	486	48%
	Graduate degree	190	19%
Income	< $40 K	394	39%
	$40 K–60 K	271	27%
	> $60 K	353	35%
	**Max**	**Mean**	**St. Dev**.
Trust	42	24.9	9.4
Digital privacy	12	10.5	1.9
Privacy behavior	21	15.4	3.3

#### Data sensitivity

In studying the relative sensitivity of respondents’ data, we include items from Milne, *et al*.‘s^15^ study of information sensitivity and willingness to provide it to various institutions for different purposes. Their study evaluated respondents’ relationships to a specific kind of social media data: the *online screen name*. Evaluating respondents’ relationships to their online screen name does not encompass all types of social media data. However, this particular social media data type, like a driver’s license number, can be a key to accessing other data about a person. Online screen names thus offer a point of comparison with similarly sensitive data types. We mirror earlier surveys’^15^ focus on the online screen name to facilitate comparisons to earlier work and other types of sensitive data. We compare the sensitivity of the online screen name to other data that can uniquely identify a specific person (e.g., driver’s license number) and to data that through linkage or in aggregate can be used to identify a specific person (e.g., race, religion, income, etc.)^61^. Sensitivity acts as a dependent variable in analyses responding to RQ1, and independent variable in analyses responding to RQ2.

#### Independent variables

To understand whether perceptions of acceptable use vary by mediating factors, we included independent variables motivated by existing literature. Based on existing research, we expected participants’ perspectives to vary based on personal characteristics like their trust in institutions generally^62^, their existing privacy practices and concerns^63^, their demographics^62,64^, and their social media use^65^. Assessing these independent variables’ effects also allows us to compare our findings with existing research on personal data sharing and sensitivity^8,11,15,27^.

### Analysis

We used generalized linear mixed models (GLMM)–specifically a cumulative link mixed model (CLMM) for ordinal outcome variables and a mixed effects logistic regression (MELR) for binary outcome variables–to analyze our survey’s results. Through the ability to include random effects, GLMMs enabled us to understand whether individual variation among participants, in addition to personal characteristics like demographics and institutional trust that we measured, impacted participants’ responses. We included a random effect for response platform (Qualtrics or MTurk) and for individual respondent. We estimated models for different combinations of independent variables, their interactions, and control variables. We include the models of best fit, determined by ANOVA, in the main text below.

### Limitations

Our survey method likely underestimates individuals’ agreement with various data uses because they may not be familiar with ways that data are used or the methods employed in analysis. For instance, prior studies of patient records^23^ and commercial access to health data^66^ found that users increased their willingness to share health data after they understood the potential for public benefit and data security measures.

We adopted the variable ‘online screen name’ from prior surveys. Had we included the specific data types we asked about in scenarios (e.g., social media posts) in the sensitivity questions, we may have gotten different results. We chose ‘online screen name’ because it enabled comparison to prior studies^15,16^, and because it is analogous to other linkable personal identifiers such as driver’s license number. Future work could examine the sensitivity of specific types of social media data.

## Data Availability

The survey data^29^ that support the findings of this study are available in Deep Blue Data with the identifier 10.7302/6vjf-av59. The University of Michigan IRB HSBS has reviewed this study and determined that it is exempt from ongoing IRB review per federal exemption category: EXEMPTION 2(i) and/or 2(ii) at 45 CFR 46.104(d) (IRB: HUM00204213).

## Code availability

Code^67^ used to perform the data preparation and analysis for this paper is available on GitHub at https://github.com/casmlab/personal-data-survey and through Zenodo with the identifier 10.5281/zenodo.6807258.
